# An Observational Study of Acquired *EGFR* T790M-Dependent Resistance to EGFR-TKI Treatment in Lung Adenocarcinoma Patients in Taiwan

**DOI:** 10.3389/fonc.2020.01481

**Published:** 2020-09-04

**Authors:** Shang-Gin Wu, Chi-Lu Chiang, Chien-Ying Liu, Chin-Chou Wang, Po-Lan Su, Te-Chun Hsia, Jin-Yuan Shih, Gee-Chen Chang

**Affiliations:** ^1^Department of Internal Medicine, National Taiwan University Hospital, National Taiwan University, Taipei, Taiwan; ^2^Department of Internal Medicine, National Taiwan University Cancer Center, National Taiwan University, Taipei, Taiwan; ^3^Division of Thoracic Oncology, Department of Chest Medicine, Taipei Veterans General Hospital, Taipei, Taiwan; ^4^Faculty of Medicine, School of Medicine, National Yang-Ming University, Taipei, Taiwan; ^5^Institute of Clinical Medicine, National Yang-Ming University, Taipei, Taiwan; ^6^Department of Pulmonary and Critical Care Medicine, Chang Gung Memorial Hospital, Taoyuan, Taiwan; ^7^Division of Pulmonary and Critical Care Medicine, Department of Medicine, Chang Gung Memorial Hospital-Kaohsiung Medical Center, Chang Gung University College of Medicine, Kaohsiung, Taiwan; ^8^Department of Internal Medicine, National Cheng Kung University Hospital, College of Medicine, National Cheng Kung University, Tainan, Taiwan; ^9^Division of Pulmonary and Critical Care Medicine, Department of Internal Medicine, China Medical University and China Medical University Hospital, Taichung, Taiwan; ^10^Division of Chest Medicine, Department of Internal Medicine, Taichung Veterans General Hospital, Taichung, Taiwan; ^11^Division of Pulmonary Medicine, Department of Internal Medicine, Chung Shan Medical University Hospital, Taichung, Taiwan; ^12^School of Medicine and Institute of Medicine, Chung Shan Medical University, Taichung, Taiwan

**Keywords:** afatinib, epidermal growth factor receptor mutation, erlotinib, gefitinib, non-small cell lung cancer, tyrosine kinase inhibitor, osimertinib

## Abstract

In Taiwan, epidermal growth factor receptor (EGFR) tyrosine kinase inhibitors (EGFR-TKIs), gefitinib, erlotinib, and afatinib are served as first-line therapy for non-small lung cell cancer (NSCLC) patients with *EGFR* sensitizing mutations. However, the majority of patients who initially respond to EGFR-TKIs, progress through acquiring *EGFR* T790M mutations (T790M), which is the most common resistant mechanism. Patients with T790M gain the opportunity of subsequent treatment with third-generation EGFR-TKI, osimertinib. This study aimed to evaluate the association between prior EGFR-TKI therapy and incidence of acquired T790M resistance in lung adenocarcinoma patients who have progressed on first/second-generation EGFR-TKI therapy. This retrospective study included lung adenocarcinoma patients who had a radiographically-confirmed progressive disease under EGFR-TKI treatment and had re-biopsy samples for T790M testing from seven medical centers in Taiwan from June 2013 to December 2018. Patients harboring *de novo* T790M or using more than one EGFR-TKI were excluded. Of the 407 patients enrolled, the overall T790M acquisition rate was 52.8%. The patients treated with gefitinib, erlotinib or afatinib had a statistically significant difference in the T790M rates (59.9, 45.5, and 52.7%, respectively; *p* = 0.037) after disease progression. Patients with common baseline *EGFR* mutations (Del-19 and L858R) (*p* = 0.005) and longer treatment duration with EGFR-TKIs (*p* < 0.001) had higher chances of T790M acquisition. Multivariate logistic regression analysis further showed that patients with common baseline *EGFR* mutations, gefitinib (compared to erlotinib) administration, and longer treatment duration with EGFR-TKIs had higher T790M incidence. There was no significant difference in the incidence of acquired T790M between different re-biopsy tissue samples or complications. In conclusion, this study showed that patients who progressed from gefitinib treatment, bearing common *EGFR* mutations, and with longer EGFR-TKI treatment duration had increased incidence of T790M acquisition and, therefore, were suitable for subsequent osimertinib treatment.

## Introduction

Lung cancer is the most commonly diagnosed cancer and the leading cause of all cancer-related mortalities in Taiwan and worldwide (1, 2). Most of the lung cancers are diagnosed at advanced or metastatic stages with lower 5-year survival rates (1). In Taiwan, 54.1% of all the newly diagnosed lung cancer cases are at stage IV, with a median survival time of 9 months (2).

Histologically, 85% of primary lung cancers are classified as non-small-cell lung cancer (NSCLC), with adenocarcinoma being the most common subtype. Somatic mutations in the *EGFR* gene are frequently found in adenocarcinomas (3, 4). The exon 19 deletion (Del-19) and exon 21 L858R (L858R) together account for 90% of the *EGFR* mutations. Other clinically relevant mutations include G719X, L861X, exon 20 insertions, etcetera (5).

Patients with NSCLC harboring *EGFR* activating mutation showed a good response to the first- (gefitinib, erlotinib) and second-generation (afatinib) of EGFR-tyrosine kinase inhibitors (TKIs), but they developed acquired resistance in about 9–13 months (6). Different mechanisms of acquired EGFR-TKIs resistance have been reported (7, 8). The most common mechanism involves the acquired *EGFR* T790M mutation, which accounts for about half of the acquired resistant cases (9, 10). To overcome T790M-mediated resistance, the third-generation EGFR-TKI, osimertinib, has shown improved median progression-free survival (PFS) in NSCLC patients with acquired T790M (11).

Tissue biopsy remains the most reliable specimen for re-biopsy analysis even considering the heterogeneous nature of tumors. Lung cytology (e.g., pleural fluid) and liquid biopsy are the less-invasive alternatives. Numerous detection platforms with high reliability and sensitivity have also been established, including direct sequencing, real-time polymerase chain reaction (qPCR), droplet digital PCR (ddPCR), next-generation sequencing (NGS), matrix-assisted laser desorption/ionization time-of-flight mass spectrometry (MALDI-TOF MS), etc. (12). Liquid biopsy testing is now increasingly used in detecting targetable alterations in NSCLC (13).

Osimertinib has not been reimbursed in Taiwan until April 2020. To identify patients for subsequent osimertinib treatment, several studies have investigated the tendency of acquired T790M under first-line EGFR-TKI treatments (14–17). In Taiwan, two single-center studies reported different rates of T790M occurrence under the treatment of the three first-line EGFR-TKIs (15, 18). Since afatinib was approved much later than the first-generation EGFR-TKIs, previous studies included fewer patients treated with afatinib compared to those treated with gefitinib and erlotinib. Consequently, we aimed to conduct a nationwide study (ARISE study) with a sufficient number of lung adenocarcinoma patients who progressed with each of the three EGFR-TKI therapies and to investigate the association between prior EGFR-TKI treatment and the incidence of acquired T790M-associated resistance.

## Materials and Methods

### Patients

This retrospective study (ARISE study) included advanced lung adenocarcinoma patients with radiographically-confirmed progressive disease after EGFR-TKI treatment. The EGFR TKIs included two first-generation drugs gefitinib and erlotinib and one second-generation drug afatinib. The participating 7 hospitals included three hospitals in northern Taiwan (National Taiwan University Hospital, Taipei Veterans General Hospital, Chang Gung Memorial Hospital Linkou Branch), two hospitals in middle Taiwan (Taichung Veterans General Hospital, China Medical University Hospital), and two hospitals in southern Taiwan (Chang Gung Memorial Hospital Kaohsiung Branch, National Cheng Kung University Hospital). Patients were ≥ 20 years old at enrollment. The documentation of confirmed *EGFR* sensitizing mutations before initiation of EGFR-TKI treatment was required. Upon disease progression from the EGFR-TKI treatment, repeat biopsy (re-biopsy) samples were obtained for the assessment of T790M mutation from June 2013 to December 2018. Some patients were reported in prior studies (15, 18).

The three EGFR-TKIs, gefitinib, erlotinib, and afatinib, have been reimbursed by the National Health Insurance (NHI) of Taiwan for patients with locally advanced or metastatic (stage IIIB/IV) lung adenocarcinoma with *EGFR* mutations for first-line treatment since 2004, 2007, and 2014, respectively. Physicians had the opportunity to choose between the three EGFR-TKIs based on patient's conditions and preferences and clinical evidence.

The information on NSCLC treatments prior to re-biopsy, including surgery, chemotherapy, radiotherapy, and TKI therapy was also collected. Chemotherapy was referred to as conventional cytotoxic agents, not including EGFR TKIs.

This study was approved by the institutional/ethical review board (IRB) of each participating medical center. Written informed consent was obtained from each patient except those with a waiver granted from the IRB of each medical center.

### Re-biopsy Specimen and T790M Detection Platform

For patients after the acquired resistance to the first/second-generation EGFR TKIs, T790M detection is a routine clinical practice. Re-biopsy specimens of primary or metastasis tumors were obtained either within the thorax (i.e., lung biopsies and pleural effusion) or out of thorax (i.e., non-lung biopsies, plasma, peritoneal fluid, and cerebrospinal fluid). The detection methods included the following: COBAS EGFR mutation test v2 (COBAS; Roche Molecular Systems Inc., New Jersey, USA), Therascreen EGFR RGQ PCR Kit (Therascreen; Scorpions & amplification refractory mutation system [ARMS], Qiagen Manchester Ltd, Manchester, UK), Beads, Emulsion, Amplification and Magnetics (BEAMing) digital PCR (dPCR) assay (BEAMing; OncoBEAM EGFR assay; Sysmex Inostics, Inc., Maryland, USA), MassARRAY genotyping (Mass; previously named SEQUENOM; Agena Bioscience, California, USA), competitive allele-specific TaqMan polymerase chain reaction (TaqMan; Life Technologies; Thermo Fisher Scientific, Inc., Massachusetts, USA), and laboratory-developed test (LDT). The laboratory-developed test for the detection of *EGFR* mutation included peptide nucleic acid locked nucleic acid sequencing (PNA-sequencing) and direct sequencing (19, 20). Each detection method was performed based on the manufacturer's instructions. All the pathology laboratories of the hospitals were certified for clinical examination by the Taiwan Society of Pathology. For the detection of T790M, the BEAMing assay had a limit of detection (LoD) <1%, whereas COBAS, Therascreen, Mass, and TaqMan tests had LoD > 1%.

### Study Endpoints

The primary endpoint was to compare the incidence of acquired T790M mutation in patients after acquired resistance to the three first-line EGFR-TKIs. This study also aimed to analyze the association of the T790M acquisition rate with baseline *EGFR* sensitizing mutations, treatment duration, and other clinical characteristics of patients.

### Statistical Analysis

The comparison of T790M acquisition from each first-line EGFR-TKI was analyzed through ANOVA or Kruskal-Wallis test for continuous data and Chi-square test for categorical data. If the sampling variability was ≤ 5, Fisher's exact test was applied. The association between clinical factors and the acquisition of T790M resistant mutation was examined using univariate and multivariate regression analyses. Time to treatment discontinuation (TTD) curves was plotted using the Kaplan-Meier method and compared using the log-rank test. All analyses were performed using SPSS software (version 22.0 for Windows; SPSS Inc., Chicago, Illinois).

## Results

### Patient Distribution and Baseline Clinical Characteristics

**Figure 1:**
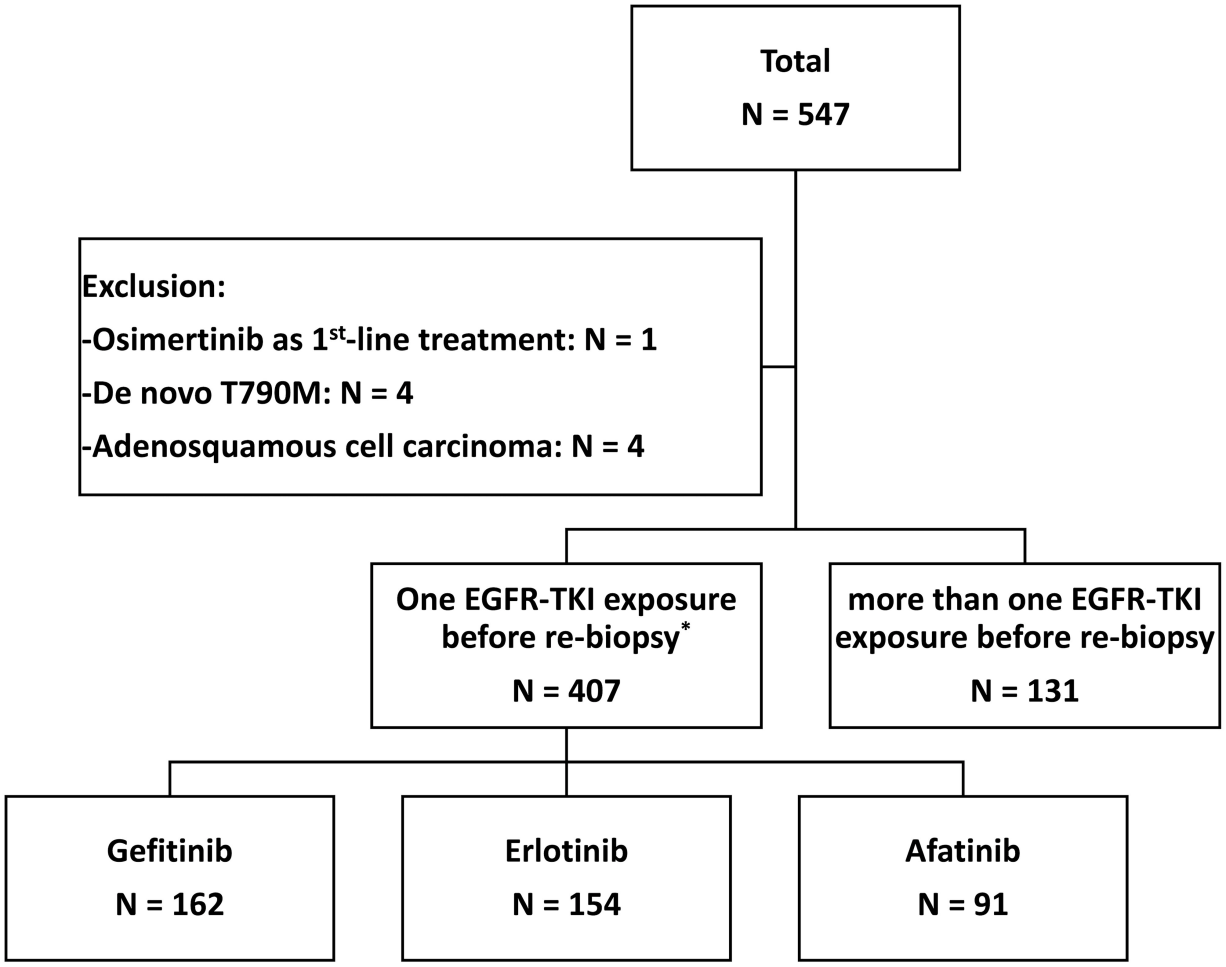
The flow chart of patient enrollment. *There were 12 patients who switched to another EGFR-TKI as a first-line treatment due to the side effects (see Supplementary Figure 1).

**Figure 2:**
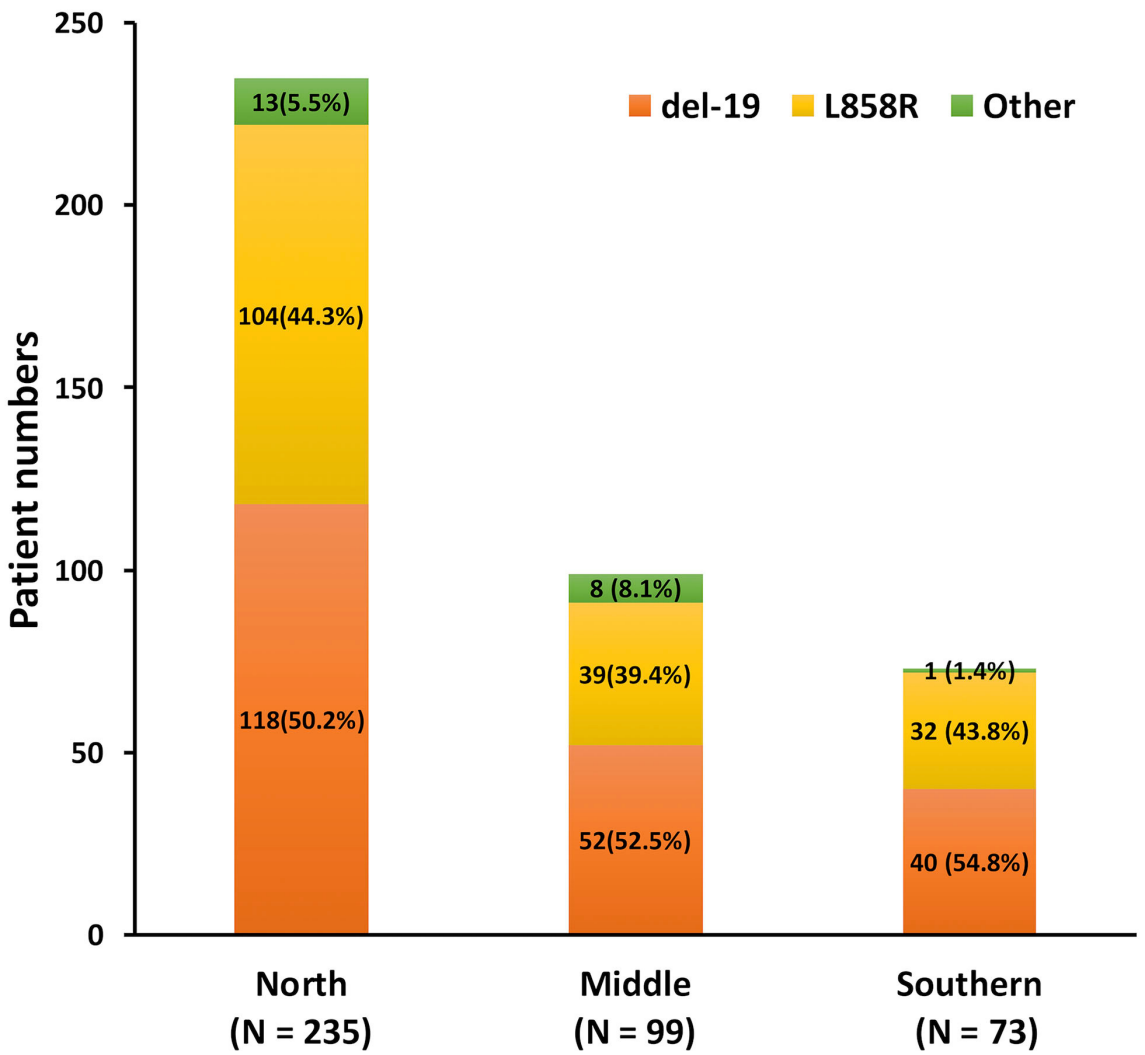
The different *EGFR* mutation types among the three areas in Taiwan. (*p* = 0.384).

From June 2013 to December 2018, 547 patients who progressed from the first-line EGFR-TKI and had re-biopsy results were recruited (Figure 1). The final analysis contained 407 lung adenocarcinoma patients after the exclusion of 1 patient with osimertinib as first-line treatment, 4 patients with *de novo* T790M before the first-line EGFR-TKI treatment, 4 patients with adenosquamous cell carcinoma, and 131 patients with more than one EGFR-TKI treatments before re-biopsy. Twelve patients who had received short-term treatment with EGFR-TKIs (7–76 days) before switching to the second or third EGFR-TKIs for 241–565 days due to adverse events, were also included into the analysis (Supplementary Figure 1). The baseline *EGFR* mutations included 210 (51.6%) Del-19, 175 (43.0%) L858R, and 22 (5.4%) other mutation types (Supplementary Table 1). There was no significant difference in different *EGFR* mutation rates, including Del-19, L858R, or uncommon *EGFR* mutation, among the three areas in Taiwan (*p* = 0.384) (Figure 2).

Among the 407 patients enrolled, 162 (39.8%), 154 (37.8%), and 91 (22.4%) used gefitinib, erlotinib, and afatinib, respectively, as first-line EGFR-TKI (Table 1). In addition, 58 (14.1%) patients were administered EGFR TKIs because of tumor recurrence after definitive surgery. There were 27 patients who received chemotherapy and 63 patients who underwent radiotherapy before EGFR TKIs administration.

**Table 1:** Clinical characteristics of EGFR-TKI-treated patients who received re-biopsy for detection of acquired T790M.

	**All patients**	**Gefitinib**	**Erlotinib**	**Afatinib**	***P***
Total	407	162 (39.8%)	154 (37.8%)	91 (22.4%)	
Age, median, years	65	67	64	63	0.002^§^
(range)	(37–96)	(41–96)	(37–93)	(37–83)	
Gender					<0.001
Female	260	121 (74.7%)	92 (59.7%)	47 (51.6%)	
Male	147	41 (25.3%)	62 (40.3%)	44 (48.4%)	
Smoking status					0.091
Never-smokers	320	138 (85.2%)	117 (76.0%)	65 (71.4%)	
Current-smokers	14	3 (1.9%)	7 (4.5%)	4 (4.4%)	
Former-smokers	73	21 (13.0%)	30 (19.5%)	22 (24.2%)	
*EGFR* mutation					<0.001
Del-19^*^	210	80 (49.4%)	71 (46.1%)	59 (64.8%)	
L858R	175	77 (47.5%)	77 (50.0%)	21 (23.1%)	
Others	22	5 (3.1%)	6 (3.9%)	11 (12.1%)	
Disease stage					0.179
Tumor recurrence	58	28 (17.3%)	22 (14.3%)	8 (8.8%)	
Advanced stage	349	134 (82.7%)	132 (85.7%)	83 (91.2%)	
Chemotherapy before re-biopsy					0.683
No chemotherapy	287	110 (67.9%)	113 (73.4%)	64 (70.3%)	
Before EGFR-TKI	27	10 (6.2%)	9 (5.8%)	8 (8.8%)	
After EGFR-TKI	93	42 (25.9%)	32 (20.8%)	19 (20.9%)	
Radiotherapy before re-biopsy					0.267
No radiotherapy	301	127 (78.4%)	106 (68.8%)	68 (74.7%)	
Before EGFR-TKI	63	18 (11.1%)	31 (20.1%)	14 (15.4%)	
After EGFR-TKI	43	17 (10.5%)	17 (11.0%)	9 (9.9%)	
Re-biopsy samples					0.004
Tumor specimens	257	115 (71.0%)	94 (61.0%)	48 (52.7%)	
Body fluid cells^†^	82	28 (17.3%)	37 (24.0%)	17 (18.7%)	
Plasma	68	19 (11.7%)	23 (14.9%)	26 (28.6%)	
T790M detection method					<0.001
LoD >1%^¶^	349	139 (85.8%)	144 (93.5%)	66 (72.5%)	
LoD <1%^π^	11	3 (1.9%)	2 (1.3%)	6 (6.6%)	
Other^ϕ^	47	20 (12.3%)	8 (5.2%)	19 (20.9%)	

§ *By Kruskal-Wallis test*.

* *Deletion in exon 19*.

† *Sampled from pleural effusion, CSF or ascites*.

¶ *COBAS EGFR Mutation Test, Scorpions & ARMS (Amplification Refractory Mutation System), competitive allele-specific TaqMan polymerase chain reaction, MassARRAY genotyping (SEQUENOM) and Therascreen test*.

π *Including BEAMing*.

ϕ *Including laboratory developed test (LDT) and others*.

*EGFR-TKI, epidermal growth factor receptor tyrosine kinase inhibitor; LoD, limit of detection*.

In the gefitinib group, there were higher proportions of females (*p* < 0.001) and patients with older age (*p* = 0.002) than in the other two groups (Table 1). Patients under gefitinib treatment also had more solid tumor specimens for re-biopsy analysis (*p* = 0.004). Patients who were treated with afatinib had fewer tumors harboring L858R (*p* < 0.001) and used more BEAMing dPCR assay and LDTs for the T790M detection (*p* < 0.001). Patients across the three EGFR-TKI groups showed no differences in the smoking status, disease stage, chemotherapy, or radiotherapy before re-biopsy.

### Development of Acquired T790M

**Figure 3:**
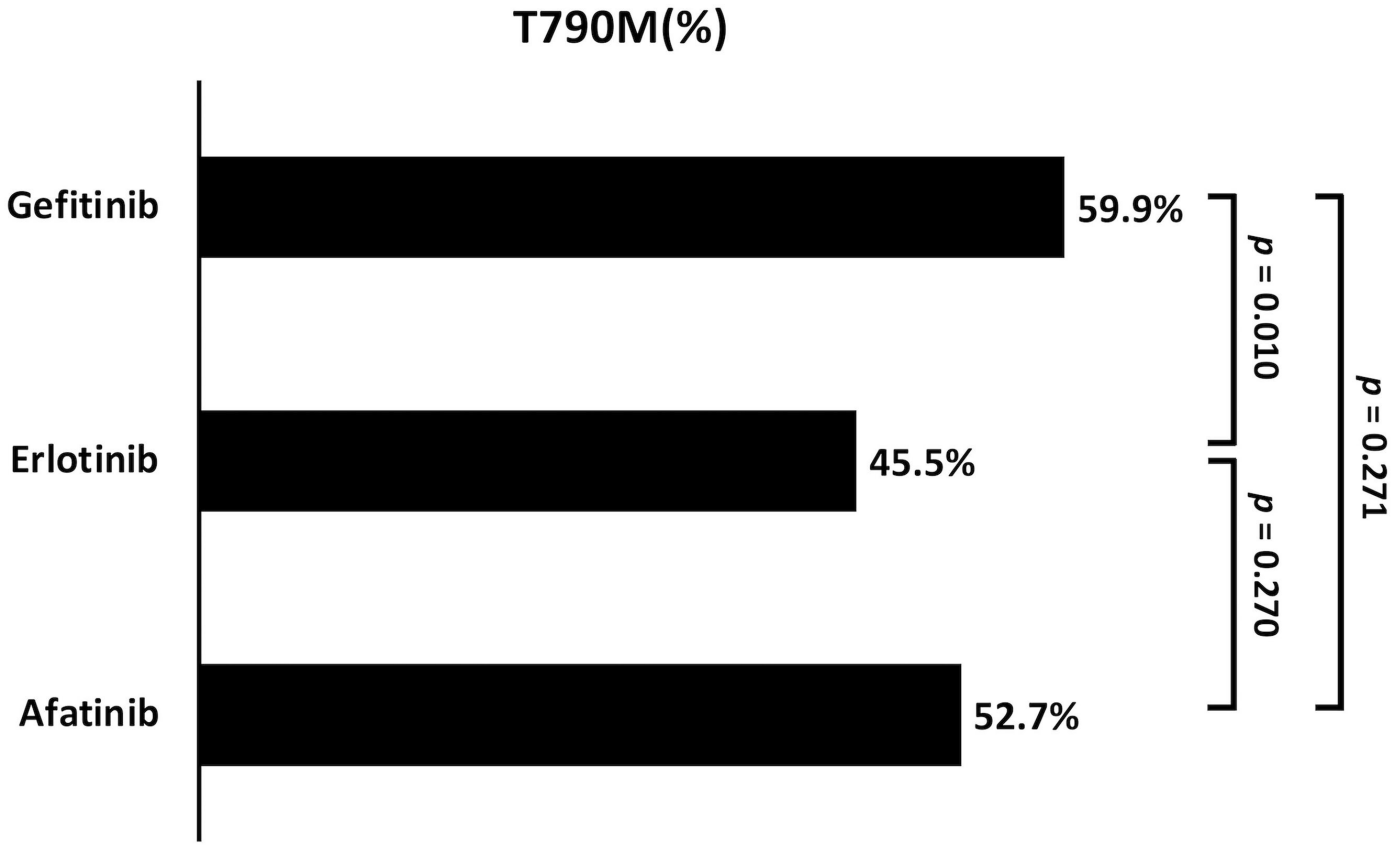
The comparison of acquired T790M incidence between the three different EGFR-TKIs (Gefitinib *vs*. Erlotinib *vs*. Afatinib: 59.9 *vs*. 45.5 *vs*. 52.7%; respectively, *p* = 0.037).

Of the 407 enrolled patients, 217 (52.8%) developed acquired T790M. The T790M acquisition rate was significantly different among patients under the three different treatments (gefitinib *vs*. erlotinib *vs*. afatinib: 59.9 *vs*. 45.5 *vs*. 52.7%, respectively; *p* = 0.037) (Table 2 and Figure 3). A higher incidence was observed in patients treated with gefitinib than in those treated with erlotinib (*p* = 0.010).

**Table 2:** Multivariate analysis of factors for acquired T790M in patients treated with EGFR-TKIs.

**Factors**	**Number of patients**	**T790M (%)**	***p***	**Multivariate analysis^#^**	
				**Odds ratio 95% CI**	***p***
Gender			0.450		
Female	260	141 (54.2)		1	
Male	147	74 (50.3)		0.75 (0.44–1.29)	0.296
Age			0.109		
≦65 y/o	210	119 (56.7)		1	
>65 y/o	197	96 (48.7)		0.74 (0.48–1.14)	0.174
Smoking status			0.265		
Never-smokers	320	170 (53.1)		1	
Current-smokers	14	10 (71.4)		3.19 (0.86–11.79)	0.083
Former-smokers	73	35 (47.9)		1.00 (0.52–1.95)	0.990
*EGFR* mutation			0.005		
Del-19^*^	210	122 (58.1)		1	
L858R	175	88 (50.3)		0.79 (0.51–1.23)	0.292
Others	22	5 (22.7)		0.18 (0.06–0.55)	0.003
EGFR-TKI			0.037		
Gefitinib	162	97 (59.9)		1	
Erlotinib	154	70 (45.5)		0.58 (0.36–0.96)	0.032
Afatinib	91	48 (52.7)		0.72 (0.40–1.33)	0.296
EGFR-TKI TTD			<0.001		
<6 mo	36	11 (30.6)		1	
6–12 mo	124	51 (41.1)		1.58 (0.68–3.65)	0.289
12–18 mo	107	62 (57.9)		3.29 (1.39–7.79)	0.007
18–24 mo	58	40 (69.0)		5.48 (2.09–14.40)	0.001
>24 mo	82	51 (62.2)		3.59 (1.46–8.84)	0.005
Chemotherapy before re-biopsy			0.356		
No chemotherapy	287	158 (55.1)		1	
Before EGFR-TKI	27	12 (44.4)		0.70 (0.29–1.70)	0.432
After EGFR-TKI	93	45 (48.4)		0.85 (0.51–1.40)	0.516
Re-biopsy samples			0.840		
Tumor specimens	257	135 (52.5)		1	
Body fluid cells^†^	82	42 (51.2)		1.08 (0.62–1.88)	0.790
Plasma	68	38 (55.9)		1.13 (0.58–2.22)	0.715
T790M detection method			0.034		
LoD >1%^¶^	349	182 (52.1)		1	
LoD <1%^π^	11	10 (90.9)		8.31 (0.95–73.03)	0.056
Other^ϕ^	47	23 (48.9)		0.73 (0.34–1.57)	0.419

* *Deletion in exon 19*.

† *Sampled from pleural effusion, CSF or ascites*.

¶ *COBAS EGFR Mutation Test, Scorpions & ARMS (Amplification Refractory Mutation system), competitive allele-specific TaqMan polymerase chain reaction, MassARRAY genotyping (SEQUENOM) and Therascreen test*.

π *Including BEAMing*.

ϕ *Including laboratory-developed test (LDT) and others*.

# *By multivariate logistic regression analyses*.

*CI, confidence interval; Del-19: deletion in exon 19; EGFR-TKI, epidermal growth factor receptor tyrosine kinase inhibitor; LoD, limit of Detection; Mo, months; TTD, Time to treatment discontinuation*.

### Incidence of Acquired T790M by Times to Treatment Discontinuation

**Figure 4:**
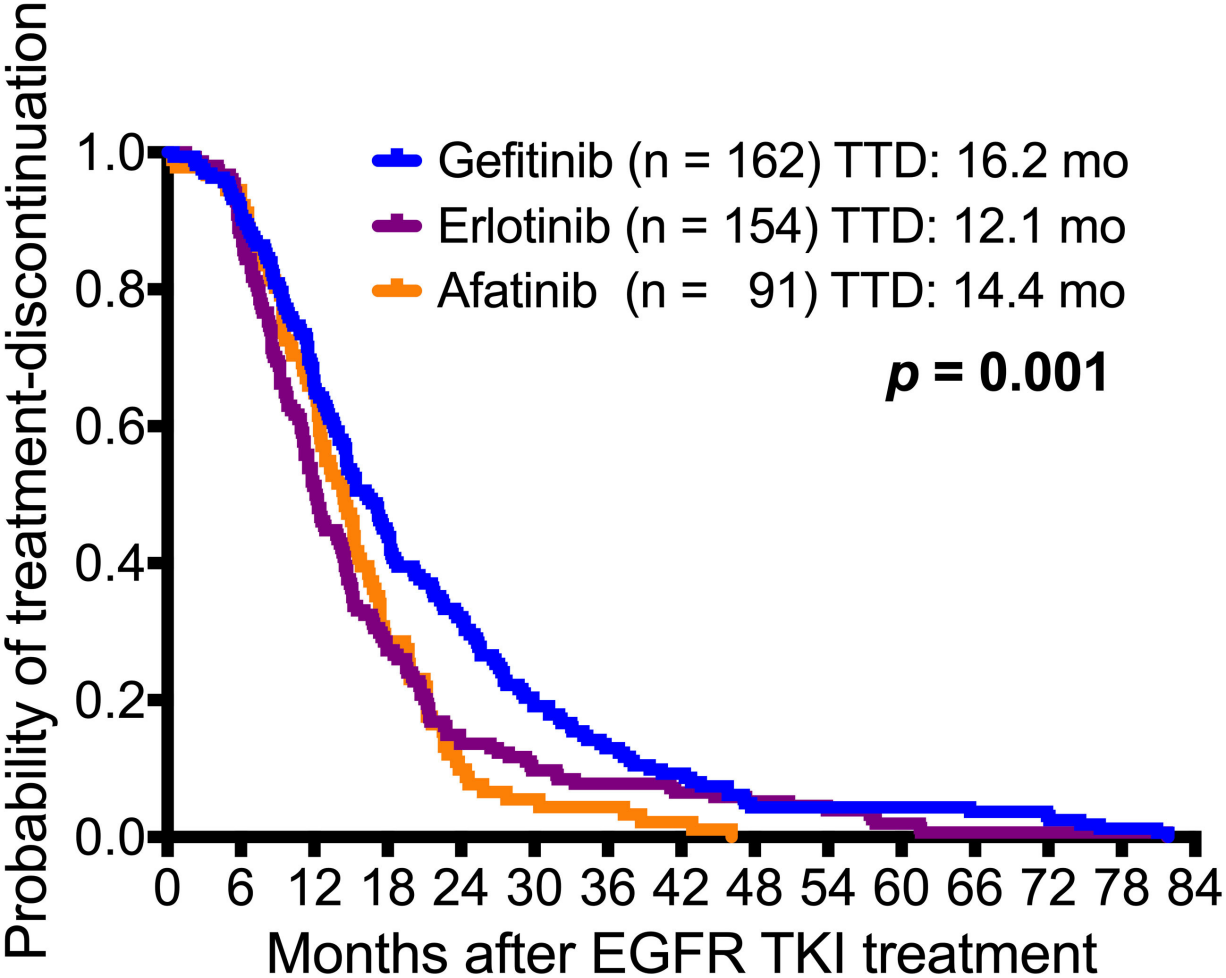
Kaplan-Meier survival curves of time to treatment discontinuation (TTD) in the three EGFR-TKI-treated adenocarcinoma patient cohorts. There was a significant difference between gefitinib (*n* = 162), erlotinib (*n* = 154), and afatinib (*n* = 91) (gefitinib *vs*. erlotinib *vs*. afatinib: 16.2 months *vs*. 12.1 months *vs*. 14.4 months, respectively; *p* = 0.001).

**Figure 5:**
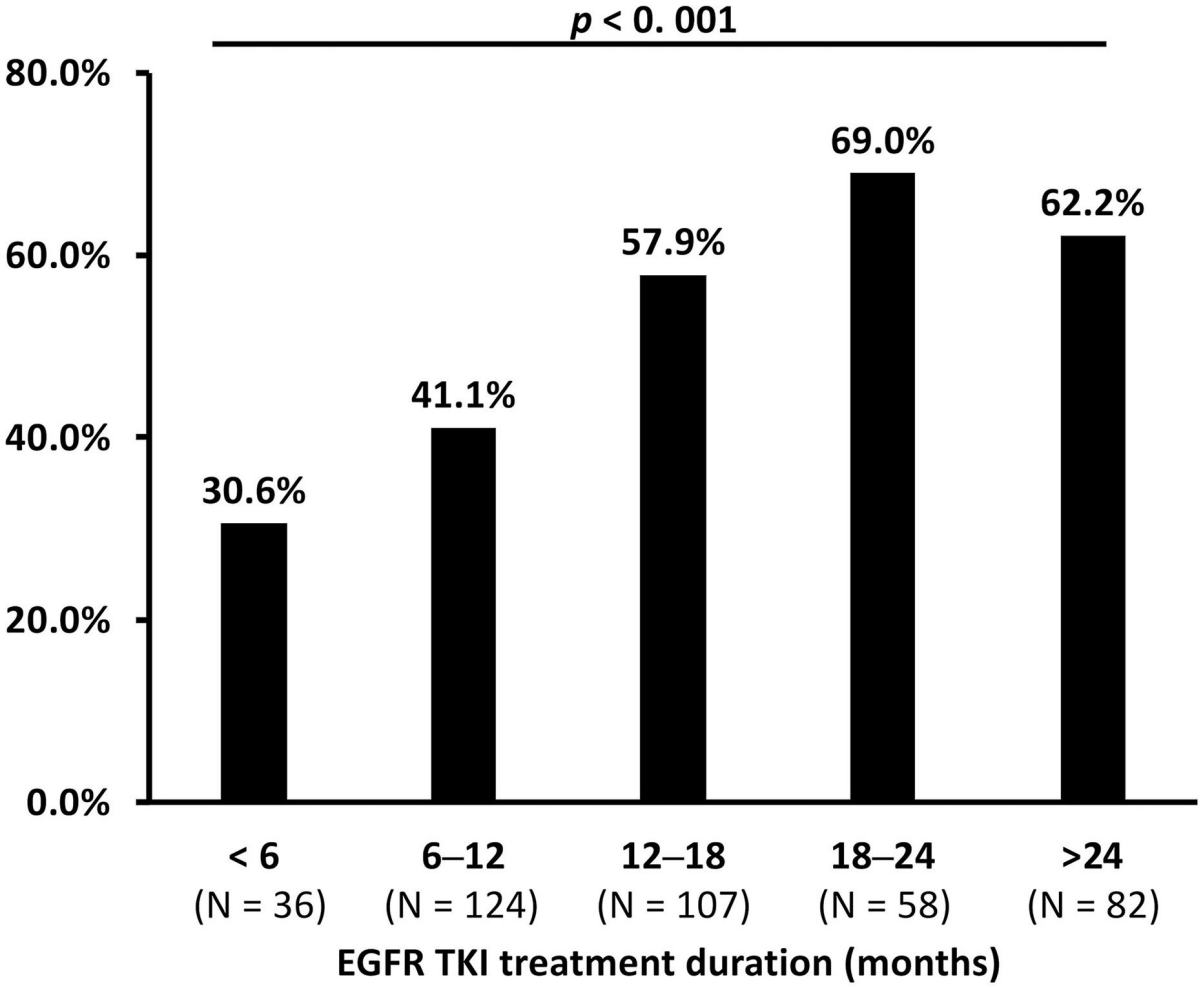
The comparison of acquired T790M incidence between different times to treatment discontinuation (TTD) of EGFR-TKIs (*p* < 0.001 by Chi-square test).

The difference in median time to treatment discontinuation (TTD) among patients who received gefitinib (16.2 months), erlotinib (12.1 months), and afatinib (14.4 months) was significant (*p* = 0.001; Figure 4). The T790M acquisition rate was analyzed by predefined TTD, and 32.1% (52 of 162) of the patients who received gefitinib had TTD of more than 24 months (Supplementary Table 2). There was a significant difference in T790M incidence among patients who had TTD of <6 months (30.6%), 6–12 months (41.1%), 12–18 (57.9%), 18–24 (69.0%) or > 24 months (62.2%) (*p* < 0.001) (Table 2 and Figure 5).

### Association of Baseline Mutations With Acquired T790M

**Figure 6:**
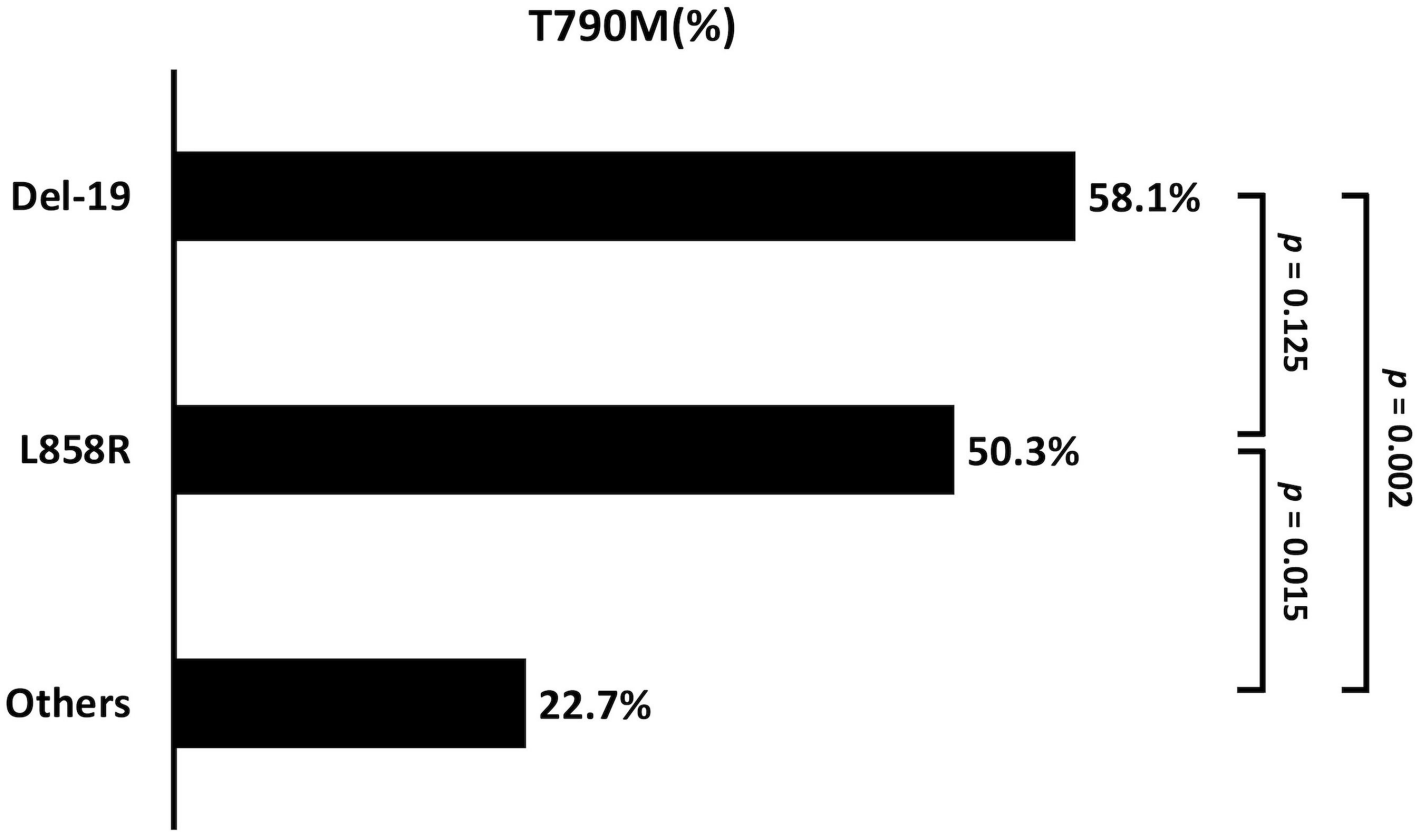
The comparison of acquired T790M incidence between the different baseline EGFR mutation types (Del-19 *vs*. L858R *vs*. Other: 58.2 *vs*. 50.3 *vs*. 22.7%; *p* = 0.004). Del-19, deletion in exon 19; ns, not significant.

Patients with baseline *EGFR* mutations of Del-19 (58.1%) or L858R (50.3%) had a significantly higher incidence of acquired T790M than those with other uncommon *EGFR* mutations (22.7%; *p* = 0.005) (Table 2 and Figure 6). There was no significant difference in the incidence of acquired T790M between patients with Del-19 and those with L858R (*p* = 0.125).

### Potential Predictive Clinical Factors for Acquired EGFR T790M

The association between patient baseline clinical characteristics and the incidence of acquired *EGFR* T790M was assessed using multivariate logistic regression analyses (Table 2). Compared with the patients who received gefitinib, those who received erlotinib had a significantly lower T790M acquisition rate (odds ratio [OR]: 0.58; 95% confidence interval [CI]: 0.36–0.96; *p* = 0.032), while patients under afatinib treatment had similar rates of acquired T790M (OR: 0.72; 95% CI: 0.40–1.33; *p* = 0.296). Patients with other uncommon baseline *EGFR* mutations were less likely to develop acquired T790M compared to those with Del-19 (OR: 0.18; 95% CI: 0.06–0.55; *p* = 0.003). In addition, compared to patients with TTD <6 months of EGFR-TKI treatments, higher T790M acquisition were seen in those with 12–18 months of TTD (OR: 3.29; 95% CI: 1.39–7.79; *p* = 0.007), 18–24 months of TTD (OR: 5.48; 95% CI: 2.09–14.40; *p* = 0.001), and > 24 months of TTD (OR: 3.59; 95% CI: 1.46–8.84; *p* = 0.005).

### Acquired T790M in Different Re-biopsy Tissue Specimens and Complications

Table 3 showed the different re-biopsy samples. The acquired T790M incidence rates were 54.4% in primary tumors, 50.0% in metastatic tumors, and 55.9% in plasma samples (*p* = 0.286). After the exclusion of plasma samples, no significant difference was noted between the re-biopsy tissues located in intra- and extra-thoracic lesions (54.4 *vs*. 43.1%; *p* = 0.101). For metastatic tumor samples, there was also no significant difference in acquired T790M incidence rates between different metastatic lesions of bone, liver, lymph nodes, or others (*p* = 0.112). It is worth noting that acquired T790M mutation was detected only in one of seven (14.3%) cerebral spinal fluids samples. Twenty-four (5.9%) patients suffered from re-biopsy complications after percutaneous needle biopsy. There were 20 pneumothoraxes, 3 haemothorax/hemopneumothorax, and one hydropneumothorax. There was no significant difference in the re-biopsy complication rate between patients with and without acquired T790M (*p* = 0.259).

**Table 3:** Acquired T790M in different re-biopsy tissue specimens and the clinical factors.

	**All patients**	**T790M(+)**	**T790M(–)**	***p***
Total	407	215 (52.8%)	192 (47.2%)	
Re-biopsy tissue specimens				0.286
Primary lung tumors	149	81 (54.4%)	68 (45.6%)	
Pleural effusions/ascites	75	41 (54.7%)	34 (45.3%)	
Cerebrospinal fluids	7	1 (14.3%)	6 (85.7%)	
Plasma	68	38 (55.9%)	30 (44.1%)	
Metastatic tumors	108	54 (50.0%)	54 (50.0%)	*0.112*^#^
*Bone*	*65*	*33*	*32*	
*Liver*	*13*	*10*	*3*	
*Lymph node*	*20*	*7*	*13*	
*Others*	*10*	*4*	*6*	
Re-biopsy tumor location^*^				0.101
Intra-thoracic	274	149 (54.4%)	125 (45.6%)	
Extra-thoracic	65	28 (43.1%)	37 (56.9%)	
Re-biopsy complication				0.259
Yes	24	10 (41.7%)	14 (58.3%)	
No	383	205 (53.5%)	178 (46.5%)	

* *Exclude plasma samples*.

# *The comparison between the differnt metatsatic tumors. The italic value demonstrated the subgroups of different metastatic tumors*.

## Discussion

This was the first nationwide study for the comparison of the T790M acquisition rate between the three first-line EGFR-TKIs, gefitinib, erlotinib, and afatinib. The study utilized a wide-range of specimen types and detection platforms and revealed a T790M acquisition rate of 52.8%. Patients who progressed from the first-line of gefitinib, erlotinib, and afatinib treatments had an incidence of T790M acquisition rate at 59.9, 45.5, and 52.7%, respectively. Patients with first-line of gefitinib treatment (compared to erlotinib), common *EGFR* mutations at baseline, and longer treatment duration had significantly higher rates of T790M. In addition, there was no significant difference in acquired T790M incidence rates neither between the different re-biopsy tissue samples nor with regard to re-biopsy-associated complications.

Identification of acquired T790M is vital since patients with T790M gain the benefit of the second-line osimertinib treatment (11). In addition, cancer cells harboring T790M mutations grow slower and have a more indolent phenotype (21). Furthermore, patients with T790M-mediated resistance tend to have longer PFS and post-progression survival compared to those without T790M (22, 23). The present study revealed a comparable T790M acquisition rate to historical data (9, 10). Some previous studies showed that the T790M acquisition rate was higher in the first-generation of EGFR-TKIs, gefitinib (50–55%), and erlotinib (38–57%), compared to that in the second-generation of afatinib (20–41%) (14–17). Our study revealed that the T790M acquisition rates were similar between gefitinib and afatinib, while it was significantly higher in patients who progressed from gefitinib than in those treated with erlotinib.

These differences in acquired T790M incidence may result from some major differences in the experimental approach when compared with previous studies (14–17). First, various assays were used for *EGFR* T790M mutation detection. The more sensitive test could detect low allele frequencies of T790M. Second, the present study enrolled a larger number of patients studied when compared to the previous single-center studies. In particular, there were more erlotinib-treated (*N* = 154) and afatinib-treated patients (*N* = 91) in the present study. Lin et al. enrolled 16 erlotinib-treated and 36 afatinib-treated patients (18). Huang et al. enrolled 13 afatinib-treated patients (15). Nosaki et al. reported 5 afatinib-treated patients (16). Third, the present study enrolled patients from multicenter in different areas of Taiwan to avoid the selection biases from in a single hospital. This study cohort was a true real-world practice in Taiwan. In addition, this study would be more generalizable to the real clinical practice of the NSCLC patient population in Taiwan. In addition, it is known that T790M mutation is located in a region where a single nucleotide polymorphism is positioned nearby the GC-rich sequences, rendering it challenging to detect and amplify.

The mechanism of acquisition T790M is still unclear. El Kadi et al. reported that deamination of the 5-methylcytosine to thymidine at position c.2369 generates the T790M change that alters TKI-binding affinity and causes resistance (24). In addition, the BELIEF trial showed that T790M at disease progression can be derived from the selection of preexisting *EGFR* T790M-positive clones or emerge *de novo* in initially negative cells (25). Advanced studies are required to explore the definite mechanism that the incidences of acquired T790M are different after variable EGFR TKIs treatment.

Our study also identified that patients with common baseline *EGFR* mutations of Del-19 and L858R had a higher T790M acquisition rate compared to those with uncommon baseline *EGFR* mutations. Lin et al. also reported that uncommon *EGFR* mutation had less secondary T790M acquisition (adjusted OR 0.14, 95% CI, 0.02–0.97; *p* = 0.047) compared with del-19 of *EGFR* mutation (18). In addition, the previous studies showed T790M was more frequent in patients with Del-19 compared to those with L858R (15, 17, 26–28), which was also observed as a trend but without statistical significance in our study. Prior studies also revealed that the incidence of T790M was higher in patients under more extended EGFR-TKI treatment before re-biopsy (14, 15, 26–29). We further stratified the data by a series of time-frame and showed that the incidence of T790M acquisition increased in accordance with the prolongation of treatment duration. We started to see the difference after 12–18 months of treatment, which is comparable to the findings of previous studies.

For body fluid or liquid biopsy, various methodologies, with high sensitivity and detection of genetic number and type alteration, are being used for the detection of *EGFR* T790M (7). Cobas EGFR Mutation Test v2 shows 61.4% of sensitivity and 78.6% of specificity (30). In addition, the sensitivity and specificity of T790M detection are 93 and 94% in the next-generation sequencing (NGS) (31, 32), 70 and 69% for beads, emulsion, amplification and magnetics (BEAMing) (33), and 77 and 63% for digital droplet polymerase chain reaction (ddPCR) (34).

There was no significant difference in the incidence of acquired T790M among different re-biopsy tissue samples, re-biopsy lesion locations (intra- *vs*. extra-thoracic), or different metastatic organs (bone, liver, lymph nodes, or others). However, CSF seems to have lower acquired T790M incidence. The prior reports showed that there was discordance in *EGFR* mutation status between primary tumor and CSF (35). It may result from low cellularity or ctDNA in the small amount of CSF or the low concentrations of the first and second generation of EGFR TKIs in CSF inadequate to drive the occurrence of T790M (36).

The main limitation of this study was the nature of the retrospective study design and potential bias. The imbalanced baseline characteristics of age, gender, *EGFR* mutation, re-biopsy sample type, and the usage of the T790M detection methods seen in this study might result from the physician's preference and the differences in technology accessibility at each medical center. Future studies utilizing randomized controlled design and unified companion diagnostic devices will help to strengthen the data. Nonetheless, the enrollment of a large population across the country and the utilization of various high-sensitive detection platforms give the strength to provide valuable information for future clinical decision-making. In addition, this study was based on a single nationality.

Osimertinib has recently been approved as the first-line treatment in patients with Del-19/L858R based on the groundbreaking results of the FLAURA trial (37). First-line osimertinib treatment reduced the risk of death by 20% compared to first-generation EGFR-TKIs and achieved a 3-year median OS of 38.6 months, which was 6.8 months longer than that achieved in the first-generation of EGFR-TKIs (37, 38). Although sequential second-line therapy of osimertinib in patients who progressed with acquired T790M attained recognized benefits, emerging evidence suggested that osimertinib as a first-line was preferable (39). In addition to the interest of reducing multiple systemic EGFR-TKI exposures that increase harmful side effects, the use of the first-line osimertinib could minimize the potential of patient loss (known as first-line treatment attrition) as a result of treatment intolerability and adverse events. It was estimated that more than 2/3 of patients might never receive clinical benefits of osimertinib due to the fact of the first-line treatment attrition and difficulties of re-biopsy and T790M detection. Despite standing as a preferable choice, the first-line osimertinib is hampered by law or price barriers in some countries. Alternatively, utilization of a first-line EGFR-TKI that is associated with a higher T790M acquisition rate remains favorable for the subsequent clinical management.

In conclusion, this study established that patients under the first-line of gefitinib treatment, bearing baseline common *EGFR* mutations, and with more than 1 year of treatment had a higher incidence rate of acquired T790M at progression, which could be managed with subsequent second-line of osimertinib treatment.

## Data Availability Statement

The raw data supporting the conclusions of this article will be made available by the authors, without undue reservation.

## Ethics Statement

The studies involving human participants were reviewed and approved by Institutional/ethical review board (IRB) of National Taiwan University Hospital, Taipei Veterans General Hospital, Chang Gung Memorial Hospital Taoyuan branch and Kaohsing Medical Center, National Cheng Kung University Hospital, China Medical University Hospital and Taichung Veterans General Hospital. Written informed consent for participation was not required for this study in accordance with the national legislation and the institutional requirements.

## Author Contributions

S-GW and J-YS: study design, literature search, and drafting of the manuscript. C-LC, C-YL, C-CW, P-LS, T-CH, J-YS, and G-CC: patient specimen collection and data collection. J-YS and G-CC: study supervision. All authors approved the final draft of the submitted manuscript.

## Conflict of Interest

S-GW has received speaking honoraria from Roche, AstraZeneca, and Pfizer. C-LC has received honoraria from AstraZeneca, Boehringer Ingelheim, and Roche. T-CH has received research grants from Eli Lilly. J-YS has received personal fees for advisory boards from AstraZeneca, Roche, Boehringer Ingelheim, Eli Lilly, Merck Sharp & Dohme, Ono Pharmaceutical, Chugai Pharmaceutical, and Bristol-Myers Squibb; speaking honoraria from AstraZeneca, Roche, Boehringer Ingelheim, Eli Lilly, Pfizer, Novartis, Merck Sharp & Dohme, Ono Pharmaceutical, Chugai Pharmaceutical, and Bristol-Myers Squibb; and travel expense from Roche, Pfizer, Merck Sharp & Dohme, Chugai Pharmaceutical, and Bristol-Myers Squibb. G-CC has received honoraria from F. Hoffmann–La Roche, Ltd, Eli Lilly and Company Oncology, AstraZeneca, Pfizer, Boehringer Ingelheim, Bristol-Myers Squibb, and Merck Sharp & Dohme. The remaining authors declare that the research was conducted in the absence of any commercial or financial relationships that could be construed as a potential conflict of interest. The handling editor declared a shared affiliation, though no other collaboration, with one of the authors P-LS.

## References

[1] Howlader N Noone A Krapcho M Miller D Brest A Yu M SEER Cancer Statistics Review, 1975-2016, National Cancer Institute. Bethesda: National Cancer Institute (2019).

[2] Wang BY Huang JY Cheng CY Lin CH Ko J Liaw YP. Lung cancer and prognosis in taiwan: a population-based cancer registry. J Thorac Oncol. (2013) 8:1128–35. 10.1097/JTO.0b013e31829ceba4 23945383

[3] Shigematsu H Lin L Takahashi T Nomura M Suzuki M Wistuba II. Clinical and biological features associated with epidermal growth factor receptor gene mutations in lung cancers. J Natl Cancer Inst. (2005) 97:339–46. 10.1093/jnci/dji055 15741570

[4] Kris M Johnson B Kwiatkowski D Iafrate A Wistuba I Aronson S Identification of driver mutations in tumor specimens from 1,000 patients with lung adenocarcinoma: the NCI's lung cancer mutation consortium (LCMC). J Clin Oncol. (2011) 29:CRA7506 10.1371/journal.pone.0040109

[5] Yeh P Chen H Andrews J Naser R Pao W Horn L. DNA-mutation inventory to refine and enhance cancer treatment (DIRECT): a catalog of clinically relevant cancer mutations to enable genome-directed anticancer therapy. Clin Cancer Res. (2013) 19:1894–901. 10.1158/1078-0432.CCR-12-1894 23344264 PMC4121886

[6] Mok TS Wu YL Thongprasert S Yang CH Chu DT Saijo N Gefitinib or carboplatin-paclitaxel in pulmonary adenocarcinoma. N Engl J Med. (2009) 361:947–57. 10.1056/NEJMoa0810699 19692680

[7] Wu SG Shih JY. Management of acquired resistance to EGFR TKI-targeted therapy in advanced non-small cell lung cancer. Mol Cancer. (2018) 17:38. 10.1186/s12943-018-0777-1 29455650 PMC5817870

[8] Camidge DR Pao W Sequist LV. Acquired resistance to TKIs in solid tumours: learning from lung cancer. Nat Rev Clin Oncol. (2014) 11:473–81. 10.1038/nrclinonc.2014.104 24981256

[9] Wu SG Liu YN Tsai MF Chang YL Yu CJ Yang PC. The mechanism of acquired resistance to irreversible EGFR tyrosine kinase inhibitor-afatinib in lung adenocarcinoma patients. Oncotarget. (2016) 7:12404–13. 10.18632/oncotarget.7189 26862733 PMC4914294

[10] Yu HA Arcila ME Rekhtman N Sima CS Zakowski MF Pao W. Analysis of tumor specimens at the time of acquired resistance to EGFR-TKI therapy in 155 patients with EGFR-mutant lung cancers. Clin Cancer Res. (2013) 19:2240–7. 10.1158/1078-0432.CCR-12-2246 23470965 PMC3630270

[11] Mok TS Wu YL Ahn MJ Garassino MC Kim HR Ramalingam SS Osimertinib or platinum-pemetrexed in EGFR T790M-positive lung cancer. N Engl J Med. (2017) 376:629–40. 10.1056/NEJMoa1612674 27959700 PMC6762027

[12] Kim E Feldman R Wistuba II. Update on EGFR mutational testing and the potential of noninvasive liquid biopsy in non-small-cell lung cancer. Clin Lung Cancer. (2018) 19:105–14. 10.1016/j.cllc.2017.08.001 28935493

[13] Aggarwal C Thompson JC Black TA Katz SI Fan R Yee SS. Clinical implications of plasma-based genotyping with the delivery of personalized therapy in metastatic non-small cell lung cancer. JAMA Oncol. (2019) 5:173–80. 10.1001/jamaoncol.2018.4305 30325992 PMC6396811

[14] Ettinger DS Wood DE Aggarwal C Aisner DL Akerley W Bauman JR NCCN guidelines insights: non-small cell lung cancer, version 1.2020. J Natl Compr Canc Netw. (2019) 17:1464–72. 10.6004/jnccn.2019.0059 31805526

[15] Huang YH Hsu KH Tseng JS Chen KC Hsu CH Su KY. The association of acquired T790M mutation with clinical characteristics after resistance to first-line epidermal growth factor receptor tyrosine kinase inhibitor in lung adenocarcinoma. Cancer Res Treat. (2018) 50:1294–303. 10.4143/crt.2017.512 29334606 PMC6192936

[16] Nosaki K Satouchi M Kurata T Yoshida T Okamoto I Katakami N. Re-biopsy status among non-small cell lung cancer patients in Japan: a retrospective study. Lung Cancer. (2016) 101:1–8. 10.1016/j.lungcan.2016.07.007 27794396

[17] Lee K Kim Y Jung HA Lee SH Ahn JS Ahn MJ Repeat biopsy procedures and T790M rates after afatinib, gefitinib, or erlotinib therapy in patients with lung cancer. Lung Cancer. (2019) 130:87–92. 10.1016/j.lungcan.2019.01.012 30885357

[18] Lin YT Chen JS Liao WY Ho CC Hsu CL Yang CY. Clinical outcomes and secondary epidermal growth factor receptor (EGFR) T790M mutation among first-line gefitinib, erlotinib and afatinib-treated non-small cell lung cancer patients with activating EGFR mutations. Int J Cancer. (2019) 144:2887–96. 10.1002/ijc.32025 30485437

[19] Chen YL Lu CC Yang SC Su WP Lin YL Chen WL. Verification of wild-type EGFR status in non-small cell lung carcinomas using a mutant-enriched PCR on selected cases. J Mol Diagn. (2014) 16:486–94. 10.1016/j.jmoldx.2014.05.007 25051378

[20] Wu CT Lin MW Hsieh MS Kuo SW Chang YL. New aspects of the clinicopathology and genetic profile of metachronous multiple lung cancers. Ann Surg. (2014) 259:1018–24. 10.1097/SLA.0000000000000385 24368645

[21] Chmielecki J Foo J Oxnard GR Hutchinson K Ohashi K Somwar R. Optimization of dosing for EGFR-mutant non-small cell lung cancer with evolutionary cancer modeling. Sci Transl Med. (2011) 3:90ra59. 10.1126/scitranslmed.3002356 21734175 PMC3500629

[22] Kuiper JL Heideman DA Thunnissen E Paul MA van Wijk AW Postmus PE. Incidence of T790M mutation in (sequential) rebiopsies in EGFR-mutated NSCLC-patients. Lung Cancer. (2014) 85:19–24. 10.1016/j.lungcan.2014.03.016 24768581

[23] Oxnard GR Arcila ME Sima CS Riely GJ Chmielecki J Kris MG. Acquired resistance to EGFR tyrosine kinase inhibitors in EGFR-mutant lung cancer: distinct natural history of patients with tumors harboring the T790M mutation. Clin Cancer Res. (2011) 17:1616–22. 10.1158/1078-0432.CCR-10-2692 21135146 PMC3060283

[24] El Kadi N Wang L Davis A Korkaya H Cooke A Vadnala V. The EGFR T790M Mutation is acquired through AICDA-mediated deamination of 5-methylcytosine following TKI treatment in lung cancer. Cancer Res. (2018) 78:6728–35. 10.1158/0008-5472.CAN-17-3370 30333118 PMC6295274

[25] Molina-Vila MA Stahel RA Dafni U Jordana-Ariza N Balada-Bel A Garzon-Ibanez M. Evolution and clinical impact of EGFR mutations in circulating free DNA in the BELIEF trial. J Thorac Oncol. (2020) 15:416–25. 10.1016/j.jtho.2019.11.023 31812754

[26] Matsuo N Azuma K Sakai K Hattori S Kawahara A Ishii H. Association of EGFR exon 19 deletion and EGFR-TKI treatment duration with frequency of T790M mutation in EGFR-mutant lung cancer patients. Sci Rep. (2016) 6:36458. 10.1038/srep36458 27811988 PMC5095551

[27] Joo JW Hong MH Shim HS. Clinical characteristics of T790M-positive lung adenocarcinoma after resistance to epidermal growth factor receptor-tyrosine kinase inhibitors with an emphasis on brain metastasis and survival. Lung Cancer. (2018) 121:12–7. 10.1016/j.lungcan.2018.04.013 29858020

[28] Liang H Pan Z Wang W Guo C Chen D Zhang J. The alteration of T790M between 19 del and L858R in NSCLC in the course of EGFR-TKIs therapy: a literature-based pooled analysis. J Thoracic Dis. (2018) 10:2311–20. 10.21037/jtd.2018.03.150 29850136 PMC5949462

[29] Kawamura T Kenmotsu H Omori S Nakashima K Wakuda K Ono A. Clinical factors predicting detection of T790M mutation in rebiopsy for EGFR-mutant non-small-cell lung cancer. Clin Lung Cancer. (2018) 19:e247–52. 10.1016/j.cllc.2017.07.002 28866043

[30] Jenkins S Yang JC Ramalingam SS Yu K Patel S Weston S. Plasma ctDNA analysis for detection of the EGFR T790M mutation in patients with advanced non-small cell lung cancer. J Thorac Oncol. (2017) 12:1061–70. 10.1016/j.jtho.2017.04.003 28428148

[31] Forshew T Murtaza M Parkinson C Gale D Tsui DW Kaper F. Noninvasive identification and monitoring of cancer mutations by targeted deep sequencing of plasma DNA. Sci Transl Med. (2012) 4:136ra68. 10.1126/scitranslmed.3003726 22649089

[32] Reckamp KL Melnikova VO Karlovich C Sequist LV Camidge DR Wakelee H. A highly sensitive and quantitative test platform for detection of NSCLC EGFR mutations in urine and plasma. J Thorac Oncol. (2016) 11:1690–700. 10.1016/j.jtho.2016.05.035 27468937

[33] Oxnard GR Thress KS Alden RS Lawrance R Paweletz CP Cantarini M. Association between plasma genotyping and outcomes of treatment with osimertinib (AZD9291) in advanced non-small-cell lung cancer. J Clin Oncol. (2016) 34:3375–82. 10.1200/JCO.2016.66.7162 27354477 PMC5035123

[34] Sacher AG Paweletz C Dahlberg SE Alden RS O'Connell A Feeney N. Prospective validation of rapid plasma genotyping for the detection of EGFR and KRAS mutations in advanced lung cancer. JAMA Oncol. (2016) 2:1014–22. 10.1001/jamaoncol.2016.0173 27055085 PMC4982795

[35] Shingyoji M Kageyama H Sakaida T Nakajima T Matsui Y Itakura M. Detection of epithelial growth factor receptor mutations in cerebrospinal fluid from patients with lung adenocarcinoma suspected of neoplastic meningitis. J Thorac Oncol. (2011) 6:1215–20. 10.1097/JTO.0b013e318219aaae 21610522

[36] Villatoro S Mayo-de-Las-Casas C Jordana-Ariza N Viteri-Ramirez S Garzon-Ibanez M Moya-Horno I. Prospective detection of mutations in cerebrospinal fluid, pleural effusion, and ascites of advanced cancer patients to guide treatment decisions. Mol Oncol. (2019) 13:2633–45. 10.1002/1878-0261.12574 31529604 PMC6887582

[37] Ramalingam SS Vansteenkiste J Planchard D Cho BC Gray JE Ohe Y. Overall survival with osimertinib in untreated, EGFR-mutated advanced NSCLC. N Engl J Med. (2020) 382:41–50. 10.1056/NEJMoa1913662 31751012

[38] Soria JC Ohe Y Vansteenkiste J Reungwetwattana T Chewaskulyong B Lee KH. Osimertinib in untreated EGFR-mutated advanced non-small-cell lung cancer. N Engl J Med. (2018) 378:113–25. 10.1056/NEJMoa1713137 29151359

[39] Gelatti ACZ Drilon A Santini FC. Optimizing the sequencing of tyrosine kinase inhibitors (TKIs) in epidermal growth factor receptor (EGFR) mutation-positive non-small cell lung cancer (NSCLC). Lung Cancer. (2019) 137:113–22. 10.1016/j.lungcan.2019.09.017 31568888 PMC7478849

