# Supplementary material for: An Observational Study of Acquired EGFR T790M-Dependent Resistance to EGFR-TKI Treatment in Lung Adenocarcinoma Patients in Taiwan
Source: Front Oncol. 2020 Sep 4;10:1481. doi: 10.3389/fonc.2020.01481 (PMC7498675; doi:10.3389/fonc.2020.01481)
Supplement: Supplementary file 1 [file Data_Sheet_1.docx]

*Supplementary Material*

Supplementary Table 1. The baseline EGFR mutation status

| ***EGFR* mutation** | **No.** | **(%)** |
| --- | --- | --- |
| **Del-19** | 210 | 51.6 |
| **L858R** | 175 | 43.0 |
| **Others** | 22 | 5.4 |
| E709V | 1 | 0.2 |
| exon 18 deletion | 1 | 0.2 |
| exon 20 insertion | 3 | 0.7 |
| G719X | 5 | 1.2 |
| G719X+L861G | 2 | 0.5 |
| G719X+S768I | 2 | 0.5 |
| L861G | 1 | 0.2 |
| L861Q | 7 | 1.7 |
| **Total** | 407 | 100% |

EGFR, epidermal growth factor receptor

**Supplementary Table 2.** Time to treatment discontinuation among different EGFR-TKIs

| **TTD** | **Gefitinib** | **Erlotinib** | **Afatinib** | **Total** |
| --- | --- | --- | --- | --- |
| **< 6 mo.** | 13 | 18 | 5 | 36 |
| **6–12 mo.** | 41 | 57 | 26 | 124 |
| **12–18 mo.** | 36 | 37 | 34 | 107 |
| **18–24 mo.** | 20 | 21 | 17 | 58 |
| **> 24 mo.** | 52 | 21 | 9 | 82 |
| **Total** | 162 | 154 | 91 | 407 |

EGFR-TKIs, epidermal growth factor receptor tyrosine kinase inhibitors; Mo, months; TTD, time to treatment discontinuation

*p* < 0.001 for patients treated with three different EGFR-TKIs.

**Supplemental Figure 1.** The treatment duration (days) of EGFR-TKIs in the 12 patients switched to another EGFR-TKIs as first-line treatment due to side effects. EGFR-TKI, epidermal growth factor receptor tyrosine kinase inhibitors
